# Supplementary material for: Difficulty with mobility among the aged in Ghana: Evidence from Wave 2 of the World Health Organization’s Study on Global Ageing and Adult Health
Source: PLoS One. 2024 Aug 27;19(8):e0290517. doi: 10.1371/journal.pone.0290517 (PMC11349201; doi:10.1371/journal.pone.0290517)
Supplement: S1 Table — (PDF) [file pone.0290517.s001.pdf]

Appendix 1

S1 Table. Individual questionnaire and coding.

The variables below were extracted from the WHO Study on Global Ageing and Adult Health (SAGE) INDIVIDUAL Questionnaire A.

**Unique Code** *(as in the WHO questionnaire)*	**Questions**	**Responses**
Q1009	Record sex of the respondent	1 MALE<br>2 FEMALE
Q1011	How old are you now?	• • • • • AGE IN YEARS
Q1012	What is your current marital status?	1 NEVER MARRIED ……._<br>2 CURRENTLY MARRIED …_<br>3 COHABITING ……………<br>4 SEPARATED/DIVORCED …_<br>5 WIDOWED………..
Q1015	Have you ever been to school?	YES<br>2 NO<br>………………………………
Q1016	What is the highest level of education that you have completed?	1 LESS THAN PRIMARY SCHOOL<br>2 PRIMARY SCHOOL COMPLETED<br>3 SECONDARY SCHOOL COMPLETED<br>4 HIGH SCHOOL(OR EQUIVALENT) COMPLETED<br>5 COLLEGE/PRE-UNIVERSITY/UNIVERSITY COMPLETED 6 POST GRADUATE DEGREE COMPLETED
Q1018	What is your background or ethnic group?	…………………..

Q1019	Do you belong to a religious denomination?	NO, NONE<br>2 BUDDHISM<br>3 CHINESE TRADITIONAL RELIGION<br>4 CHRISTIANITY (INCLUDING ROMAN CATHOLIC, PROTESTANT, ORTHODOX, OTHER)<br>5 HINDUISM<br>6 ISLAM<br>7 JAINISM<br>8 JUDAISM<br>9 PRIMAL INDIGENOUS (INCLUDING AFRICAN TRADITIONAL AND DIASPORIC)<br>10 SIKHISM<br>87 *OTHER , SPECIFY:*<br>97 REFUSED
Q1020	Have you always lived in this village/town/city?	YES …………_<br>2 NO
Q1501	Have you ever worked?	1. YES ………<br>2 NO
Q2000	In general, how would you rate your health today?	1. Very good<br>2 Good<br>3 Moderate<br>4 Bad<br>5 Very bad
Q2001	Overall in the last 30 days, how much difficulty did you have with work or household activities?	1 None<br>2 Mild<br>3 Moderate<br>4 Severe<br>5 Extreme/cannot do
**Mobility**	Overall in the last 30 days, how much difficulty did you have …	
Q2002	……..with moving around?	NONE<br>MILD<br>MODERATE<br>SEVERE<br>EXTREME /CANNOT DO
Q2003	……in vigorous activities ('vigorous activities' require hard physical effort and cause large increases in breathing or heart rate)?	NONE<br>MILD<br>MODERATE<br>SEVERE<br>EXTREME /CANNOT DO
Q2504	What is your height in centimeters?	……centimetres
Q2505	What is your weight in kilograms?	….. kilograms
Q3005	In the past, did you ever smoke tobacco or use smokeless tobacco daily?	1 Yes<br>2 No
Q3007	Have you ever consumed a drink that contains alcohol (such as beer, wine, spirits, etc.)?	1 YES<br>2 NO, NEVER

Q3016	Does your work involve vigorous-intensity activity that causes large increases in breathing or heart rate, [like heavy lifting, digging or chopping wood] for at least 10 minutes continuously?	1 YES<br>2 NO
Q4001	Have you ever been diagnosed with/told you have arthritis (a disease of the joints, or by other names rheumatism or osteoarthritis)?	1 YES<br>2 NO …………………
Q2007	Overall in the last 30 days, how much of bodily aches or pains did you have?	None<br>Mild<br>Moderate<br>Severe<br>Extreme/cannot do
Q2016	Overall in the last 30 days, how much of a problem did you have with sleeping, such as falling asleep, waking up frequently during the night or waking up too early in the morning	None<br>Mild<br>Moderate<br>Severe<br>Extreme/cannot do
Q4022	Have you ever been diagnosed with diabetes (high blood sugar)?	1 YES<br>2 NO
Q4040	Have you ever been diagnosed with depression?	1 YES<br>2 NO
Q4060	Have you ever been diagnosed with high blood pressure (hypertension)?	1 YES<br>2 NO
Q2024	In the last 30 days, how much difficulty did you have in seeing and recognising an object at arm's length (for example, reading)?	None<br>Mild<br>Moderate<br>Severe<br>Extreme/cannot do
Q4069	In the last 12 months, have you been involved in a road traffic accident where you suffered from bodily injury?	Yes<br>No
