# Supplementary material for: Difficulty with mobility among the aged in Ghana: Evidence from Wave 2 of the World Health Organization’s Study on Global Ageing and Adult Health
Source: PLoS One. 2024 Aug 27;19(8):e0290517. doi: 10.1371/journal.pone.0290517 (PMC11349201; doi:10.1371/journal.pone.0290517)
Supplement: S2 Table — (PDF) [file pone.0290517.s002.pdf]

S2 Table. Multicollinearity test results

Variable	VIF	Tolerance	R-Squared
Age group	1.35	0.739	0.260
Gender	1.47	0.681	0.319
Marital status	1.52	0.658	0.342
Had formal education	1.15	0.866	0.134
Perceived health status	1.28	0.782	0.219
Difficulty with household activities	1.58	0.632	0.368
Sleep problems	1.42	0.704	0.296
Bodily pains	1.64	0.610	0.390
Difficulty with sight	1.24	0.806	0.194
Engage in vigorous activities	1.15	0.866	0.134
**Mean VIF**	**1.38**		

Source: Computed from 2014/2015 WHO's SAGE Wave – 2
