# Supplementary material for: Difficulty with mobility among the aged in Ghana: Evidence from Wave 2 of the World Health Organization’s Study on Global Ageing and Adult Health
Source: PLoS One. 2024 Aug 27;19(8):e0290517. doi: 10.1371/journal.pone.0290517 (PMC11349201; doi:10.1371/journal.pone.0290517)
Supplement: Attachment — Submitted filename: Rebuttal Letter - PloS One.docx [file pone.0290517.s003.docx]

**Rebuttal Letter – Manuscript PONE-D-23-02149 resubmitted as PONE-D-23-22220**

Dear Dr Boakye,

Thank you for submitting your manuscript to PLOS ONE. After careful consideration, we have decided that your manuscript does not meet our criteria for publication and therefore must be rejected.

I am sorry that we cannot be more positive on this occasion, but hope that you appreciate the reasons for this decision.

Kind regards,

Nestor Asiamah, PhD

Academic Editor

PLOS ONE

Additional editor comments:

Dear Author(s),

Thanks for submitting your manuscript to PLOS ONE. However, the manuscript is not formatted according to PLOS ONE’s guidelines; hence, I’m returning the manuscript to you. Please format the manuscript appropriately and resubmit it.

Response: Thank you very much for the feedback on the manuscript. Please the authors have revised thoroughly the manuscript and formatted it according to PLOS ONE’s guidelines. We hope that this manuscript has now met the PLOS ONE’s guidelines and will be considered.

Thank you.
