# Supplementary material for: Difficulty with mobility among the aged in Ghana: Evidence from Wave 2 of the World Health Organization’s Study on Global Ageing and Adult Health
Source: PLoS One. 2024 Aug 27;19(8):e0290517. doi: 10.1371/journal.pone.0290517 (PMC11349201; doi:10.1371/journal.pone.0290517)
Supplement: Attachment — Submitted filename: Response to Reviewers.docx [file pone.0290517.s004.docx]

Dear Reviewers,

We are grateful to the reviewers for squeezing time out of their busy schedules to review this manuscript. In this rebuttal letter (point-by-point response), we have addressed each comment raised by the reviewers and highlighted the changes in the revised manuscript; yellow highlight for reviewer 1 and turquoise highlight for reviewer 2. We believe that the manuscript has improved substantially and we hope that it would now merit publication.

Reviewer #1: Research idea to generate evidence for geriatric care is good. Authors have skills to do secondary data analysis. However, there are conceptual issues in the analysis:

Response: Thank you for acknowledging that generating evidence for geriatric care is good coupled with commenting on the authors expertise. We have revised the conceptual issues in the analysis as well.

1. Dependent variable is construed by merging two questions: one on mobility difficulty and the other in vigorous activity. This is likely to inflate the mobility problem. It is not expected that elderlies will not face problems for the vigorous activities. Therefore, authors should either drop the second variable or do separate analysis for the same.

Response: Thank you for the comment. The authors have now dropped the second variable and revised the manuscript accordingly.

Secondly it is not best idea to merge mild problem with severe problem and treat in one category. Oversimplification of data set for analysis sometimes lead to loss of information. Mild problems can be merged with no problems also. Or some scoring system can be devised. It will be interesting to know how these problems increase with age. (including the severity)

Response: Thank you for the comment. This categorization was adopted to determine those who experienced any difficulty with mobility rather than the degree of difficulty. Additionally, this categorization followed a previous study in Ghana on functional difficulties among the aged. (Awuviry-Newton K, Wales K, Tavener M, Kowal P, Byles J. Functional difficulties and toileting among older adults in Ghana: Evidence from the World Health Organization Study on global AGEing and adult health (SAGE) Ghana Wave 1. Ageing Soc. 2021;53–75.)

2. Predictors also need relook. Pains and discomfort can be merged into one predictor.

Response: Thank you for the comment: Bodily discomfort have been dropped from the predictors.

Vigorous activity is also included in predictor and it was also there in the dependent variable. This may be dropped from predictor.

Response: Thank you for the comment: Vigorous activity is now dropped from the dependent variables and hence considered as independent variable this time around.

Authors can also analysis association with smoking and alcohol etc for which data is available to them.

Response: Smoking and alcohol were considered as independent variables. However, they were not significant at 5% alpha level in the chi-square test and hence not considered in the regression model.

Stroke will lead to mobility problem. It can be dropped as predictor in study related to ageing.

Response: Thank you for the comment, stroke is now dropped as a predictor in the revised manuscript

3. Authors to look to typo errors. Data table for self-reported health problem is misreported.

Response: Thank you for the comment: Changes made appropriately

4. Collinearity: How have authors tested collinearity for categorical predictors and what cutoff of VIF was used for decision.

Response: Thank you for the comment: Collinearity diagnostics test was conducted on the independent variables. The cut-off point for the VIF was 10.0. The results indicated no evidence of collinearity of the independent variables (please see S2 Table).

Response:

Reviewer #2: Abstract and background well-articulated. Methods as a title is required in line 107, before describing the data collection and analysis.

Response: Thank you for the comment: Revision is made appropriately in the revised manuscript.

Dependent variables
The quotes required for the responses should not be doubled. I would suggest revising and using the single quote.

Thank you for the comment: the dependent variable has been revised and a single question or quote is now used.

Independent variables
The sentence is too long to list 18 independent variables in one sentence. Please try to split and describe them with conjunction.

Response: Thank you for the comment, the sentence has been revised and divided into two sentences.

Similar comment from line 137-145, it is a long sentence. In addition, single quotes are required for the possible responses.

Response: The changes have been made appropriately. Thank you.

Results
I would suggest describing the number and per cent: N (%). It is confusing for the reader that nearly 1/3, 2/3, ¾, nearly half… in all your description.

Response: Thank you for the comment. The authors do understand describing frequencies and percentage as suggested. However, we do believe presenting the percentages (%) together with the phrases used for the frequencies perfectly describes the health problem rather than mere numbers.

The author should be selective in describing the key demographics. It is not necessary to describe all variables in the table. Similar comment to the description in figure 1.

Response: Thank you for the comment. The authors have taken note of that and revised the manuscript as such.

The description in table 2 and table 3 is not clear, and a bit confusing. The author simply listed and compared the variables. What is the result indicating, what does the association mean? All this should be revised.

Response: Thank you for the comment. Even though, Tables 2 and 3 all determine the association between the dependent and independent variables, there is a strict difference between the two tables. Table 2 only determines whether there is an association between the dependent and independent variables at 5% alpha level (this was utilized as criteria to select variables into the regression model or Table 3). Only variables significant at 5% alpha level in Table 2 will be introduced to Table 3 for the regression model.

However, the Table 3 further determines the direction of the association (whether negative or positive) and the degree of the association, which Table 2 cannot estimate. Additionally, in regression model, we always compare the significant category of a variable to the baseline, and that was how the descriptions were written.

Discussion
The first two paragraphs are repeating the results. In discussion, the author is expected to interpret the results.

Response: The first paragraph is not a repetition of the results. However, the authors reiterated the key findings from the study before commencing the discussion.

I also have seen some irrelevant words such as ‘interestingly’ in your discussion. Please try to revise and discuss the key findings of your study.

Response: The word “interestingly” has been deleted and the discussion revised as such.

Declaration
Why is ethical approval written twice? If this is the place to write, it is not necessary to mention in the method section.

Response: Thank you for the comment. The ethics statement is now written once at the methods section.

Why is not applicable consent for publication?

Response: Thank you for the comment: the authors indicated “not applicable” because they were not directly involved in data collection from patients but relied on secondary data for the study.

References
The list of references should be consistent and well written. Please have a look at references such as reference number 20, 27 and the last references.

Response: Changes effected accordingly.
