# Supplementary material for: Difficulty with mobility among the aged in Ghana: Evidence from Wave 2 of the World Health Organization’s Study on Global Ageing and Adult Health
Source: PLoS One. 2024 Aug 27;19(8):e0290517. doi: 10.1371/journal.pone.0290517 (PMC11349201; doi:10.1371/journal.pone.0290517)
Supplement: Attachment — Submitted filename: Rebuttal plos one 06072024.docx [file pone.0290517.s005.docx]

Review Comments to the Author

Reviewer #2: Abstract
The first sentences of the abstract should be revised. The flow of the sentence is not clear. I would suggest starting from the second sentence.

Response: Thank you for the comment. Please the first sentence has been revised.

Methods
What is the standard age for the aged population? Why do the authors include participants aged 50 years and over? What does the aged in Ghana mean?

Response: Thank you for the comment. There is no standard or acceptable definition for the aged in Ghana. By proxy, we rely on the categorization by the Ghana Statistical Service (≥60 years) which also strongly relies on the age for pension or retirement in Ghana (which is still relative as some people go on voluntary pension at age 55 years). We included the participants aged 50 years and over because the WHO SAGE study classified them as aged population (see these studies: Kowal P, Naidoo N, Chatterji S. WHO’s Study on Global AGEing and Adult Health (SAGE). Encycl Gerontol Popul Aging. 2019;1–6 and Gildner TE, Liebert MA, Kowal P, Chatterji S, Snodgrass JJ. Associations between sleep duration, sleep quality, and cognitive test performance among older adults from six middle-income countries: results from the Study on Global Ageing and Adult Health (SAGE). J Clin Sleep Med. 2014 Jun 15;10(6):613-21. doi: 10.5664/jcsm.3782. PMID: 24932140; PMCID: PMC4031401).

Additionally, studies conducted in Ghana also classified the aged population by including participants aged 50 years and over. (See studies; Nutakor JA, Dai B, Zhou J, Larnyo E, Gavu AK, Asare MK. Association between socioeconomic status and cognitive functioning among older adults in Ghana. Int J Geriatr Psychiatry. 2021;36(5):756–65., Awuviry-Newton K, Wales K, Tavener M, Kowal P, Byles J. Functional difficulties and toileting among older adults in Ghana: Evidence from the World Health Organization Study on global AGEing and adult health (SAGE) Ghana Wave 1. Ageing Soc. 2021;53–75, and Amegbor PM, Braimah JA, Adjaye-Gbewonyo D, Rosenberg MW, Sabel CE. Effect of cognitive and structural social capital on depression among older adults in Ghana: A multilevel cross-sectional analysis. Arch Gerontol Geriatr [Internet]. 2020;89(September 2019):104045. Available from: https://doi.org/10.1016/j.archger.2020.104045). Therefore, the authors had substantial information or evidence to include participants 50 years and above as the aged in Ghana. This notwithstanding, we have included it as a limitation in the study.

Conclusion: It is a repetition of results especially the first sentences. It will be more relevant to include the summary of the findings, the implication and recommendation.

Response: The conclusion has been revised appropriately. Thank you.

Dependent variables
As I mentioned in the previous comments, I have not seen any change with the quotes. I believe a single quote should be used for the responses. The authors said it is revised. However, I have not seen any revised version here.

Response: Thank you for the comment. The authors are now using a question which was used to ask participants about their difficulty with mobility. In the previous submission, difficulty in mobility and difficulty in engaging in vigorous activity were combined (as dependent variable), upon the comments from the reviewers, the authors recomputed the dependent variable and run the entire analysis again. We believe that has addressed the comment raised.

Independent variables
Similar comments with the quotes to the independent variables starting from line 144 to 155. Here the author revised the long sentences which are important. However, the author used additionally as a conjunction round three times. It will be more relevant to use synonyms rather than repeating the same word.

Response: Thank you for the comment. The authors have revised the quotes related to the independent variables.

Results
The description of results is not revised. The Authors simply describe one third, nearly… repeatedly. It is relevant to use different types of description rather than repetition of words. I would again suggest the number and percent N (%). Similar comments to all types of description.

Response: Thank you for the comment. The authors have revised all the descriptions of the results and taken your suggestion.

Reviewer #3: The manuscript has been suitably updated as advised by the reviewers. SAGE is a benchmark longitudinal study in the field of ageing in community.

Response: Thank you for re-reviewing the manuscript and also acknowledging that all the comments raised have been addressed and the manuscript is suitably updated.
